# Plastoquinone redox status influences carboxysome integrity via a RpaA‐ and reactive oxygen species‐dependent regulatory network

**DOI:** 10.1111/tpj.70480

**Published:** 2025-09-19

**Authors:** María Santos‐Merino, Lauri Nikkanen, Emmanuel J. Kokarakis, Yagut Allahverdiyeva, Daniel C. Ducat

**Affiliations:** ^1^ MSU‐DOE Plant Research Laboratory Michigan State University East Lansing Michigan 48824 USA; ^2^ Laboratory of Molecular Plant Biology, Department of Life Technologies University of Turku Turku 20014 Finland; ^3^ Department of Microbiology and Molecular Genetics Michigan State University East Lansing Michigan 48824 USA; ^4^ Department of Biochemistry and Molecular Biology Michigan State University East Lansing Michigan 48824 USA

**Keywords:** Regulator of Phycobilisome Association A, carboxysome, plastoquinone pool, redox status, integrity

## Abstract

Carboxysomes are bacterial microcompartments that encapsulate Rubisco and are a core component of the cyanobacterial carbon concentration mechanism (CCM). While carboxysome number, size, and spatial organization vary in different environmental conditions (CO_2_, light availability, redox state, temperature, and light quality), the molecular mechanisms underlying this potentially adaptive process remain elusive. Herein, we observe that mutants of the circadian rhythm/metabolism factor, Regulator of Phycobilisome Association A (RpaA), exhibit a striking breakdown of carboxysomes under certain environmental conditions. We find that conditions leading to overreduction of the plastoquinone (PQ) pool (mixotrophic growth, high irradiance, or chemical inhibition of electron transfer from PQ to the cytochrome *b*_*6*_ *f* complex) are accompanied by an elevated generation of reactive oxygen species (ROS) and correlate with the loss of carboxysome integrity. Carboxysome breakdown is reversed by environmental conditions or chemical inhibitors that prevent PQ overreduction and accompanying ROS generation. Taken together, our data support a novel link between the redox status of the PQ pool and carboxysome integrity. Our results have implications for the fundamental understanding of cyanobacterial energy‐balancing pathways and may indicate new research directions for understanding how the carboxysome is remodeled in response to changing environments.

## Significance Statement

We identified a dramatic remodeling of carboxysomes under conditions that lead to overreduction of the plastoquinone pool of the photosynthetic electron transport chain. This overreduction was accompanied by an accumulation of reactive oxygen species that were exacerbated in RpaA‐deficient mutants, leading to loss of carboxysome integrity. Our results suggest that the plastoquinone pool – and thereby the light reactions – are linked to dynamic carboxysome changes via a regulatory network that involves reactive oxygen species and/or RpaA.

## INTRODUCTION

Cyanobacteria and other photosynthetic organisms employ a variety of adaptive pathways to contend with variability in light (Labiosa et al., 2006). In photosynthetic linear electron flow, electrons extracted from water are transferred from photosystem II (PSII) to photosystem I (PSI) via a series of electron carriers, including the membrane‐soluble plastoquinone (PQ) pool. Spikes in illumination can result in an overreduced photosynthetic electron transport chain and lead to the generation of toxic reactive oxygen species (ROS) (Calzadilla & Kirilovsky, 2020; Foyer, 2018). A proportional increase in ATP/NADPH requirements can also create an energy imbalance, leading to the reduction or oxidation of the electron transport chain and the PQ pool (Fan et al., 2021; Fujita et al., 1987; Sunil et al., 2013). State transitions (Calzadilla & Kirilovsky, 2020) and photoprotective systems (such as orange carotenoid protein or flavodiiron proteins) (Kirilovsky & Kerfeld, 2016; Nikkanen et al., 2021) are well‐studied processes by which cyanobacteria attempt to rebalance the redox status of the photosynthetic electron transport chain (pETC) in the short term. Unbalanced photosynthetic activity can lead to the formation of ROS, typically regarded as a dangerous byproduct, although some species (i.e., hydrogen peroxide [H_2_O_2_]) have well‐established signaling roles (Mironov et al., 2019).

In addition to short‐term changes in light availability, day/night cycles introduce irradiance changes that are anticipated by the well‐characterized cyanobacterial circadian rhythm machinery (Cohen & Golden, 2015; Diamond et al., 2015; Dong et al., 2010; Martins et al., 2018; Pattanayak et al., 2014; Taton et al., 2020; Yang et al., 2010). The cyanobacterial circadian clock consists of a core oscillator, KaiC, which undergoes cyclic rounds of phosphorylation and dephosphorylation that are programmed by upstream regulators connected to light reactions via the PQ pool and has output functions controlled by the two‐component system proteins SasA and RpaA (Regulator of Phycobilisome Association A) (Ivleva et al., 2006; Kim et al., 2012, 2020; Nakajima et al., 2005; Takai et al., 2006). RpaA is an OmpR‐type response regulator that acts as a master regulator of the circadian clock output pathways, that drive global rhythms of gene expression and gating of cell division (Ashby & Mullineaux, 1999; Dong et al., 2010; Markson et al., 2013). Additionally, other functions have been subscribed to RpaA beyond its roles in entraining the circadian clock and controlling clock output (Diamond et al., 2015, 2017; Iijima et al., 2015; Puszynska & O'Shea, 2017). For example, it has been proposed that RpaA may be more directly involved in regulating enzymes of core metabolism to influence carbon partitioning (i.e., towards glycogen storage vs. downstream metabolism) (Puszynska & O'Shea, 2017), as well as directing the energy from electron‐energy transfer pathways to downstream carbon metabolism (Johnson et al., 2024).

Captured light energy is utilized by downstream metabolism, especially the carbon fixation reactions predominantly confined to carboxysomes. Carboxysomes are bacterial microcompartments comprised of a proteinaceous shell that encapsulates Rubisco and which are a central component of the cyanobacterial carbon concentration mechanism (CCM) (Burnap et al., 2013). The primary function of the carboxysome is to maximize the carbon‐fixing capacity of Rubisco by defining a microenvironment that maximizes inorganic carbon availability and simultaneously minimizes photorespiratory flux (Long et al., 2007). Carboxysomes respond to environmental changes by adjusting in number, size, and spatial organization based on CO_2_, light availability, redox state, temperature, and light quality (Lucius & Hagemann, 2024; Rillema et al., 2021; Rohnke et al., 2018; Sun et al., 2016, 2019). Their positioning and carbon fixation capacity also vary with diurnal cycles and carbon demand (Singh et al., 2022; Sun et al., 2020). For example, in the model cyanobacterium *Synechococcus elongatus* PCC 7942 (*S. elongatus*), an increase in irradiance is generally correlated with increased carboxysome number (Sun et al., 2016), suggestive of a regulatory link between the availability of light energy and cellular investment in carbon fixation machinery. Similarly, we have recently shown that carboxysome numbers increase in *S. elongatus* strains when heterologous metabolic pathways are expressed that increase the cellular metabolic “demand” (Santos‐Merino, Singh, & Ducat, 2021; Singh et al., 2022). Therefore, the “upstream” processes of photosynthetic light reactions appear to be integrated with the “downstream” energy demands of the cell for carbon fixation and metabolism, although regulatory mechanisms that accomplish this are poorly understood (Santos‐Merino, Singh, & Ducat, 2021).

We recently conducted a screen of all known two‐component regulatory proteins of *S. elongatus* with the goal of identifying potential cyanobacterial networks important for achieving energy balance between photosynthetic energy harvesting and integrated metabolic demand (Santos‐Merino et al., 2024). Briefly, our approach used a heterologous metabolic pathway (sucrose secretion) that can be experimentally activated to significantly draw upon primary products of photosynthesis. Activation of the sucrose secretion pathway has been previously shown to lead to a range of photosynthetic changes, including increased carboxysome number, increased CO_2_ fixation rates, increased oxygen evolution, and reduced acceptor side limitation of PSI activity (Abramson et al., 2016; Santos‐Merino, Torrado, et al., 2021; Singh et al., 2022). Our screen for regulatory proteins involved in this process implicated RpaA, ManS, CikB, and NblS as leading factors important in coupling changes in metabolic demand in the cell to upstream enhancements in the flux of photosynthetic processes. Herein, we report potential mechanisms that link RpaA function to the control of the organization of the carboxysome in response to ROS signals derived from an overreduced pETC. Our results have implications for fundamental understanding of cyanobacterial energy‐balancing pathways and may provide insight into how the carboxysome is remodeled in response to changing environments and the utilization of central carbon metabolic intermediates.

## RESULTS

### Remodeling carbon fixation machinery following sucrose secretion requires *rpaA*

**Figure 1:**
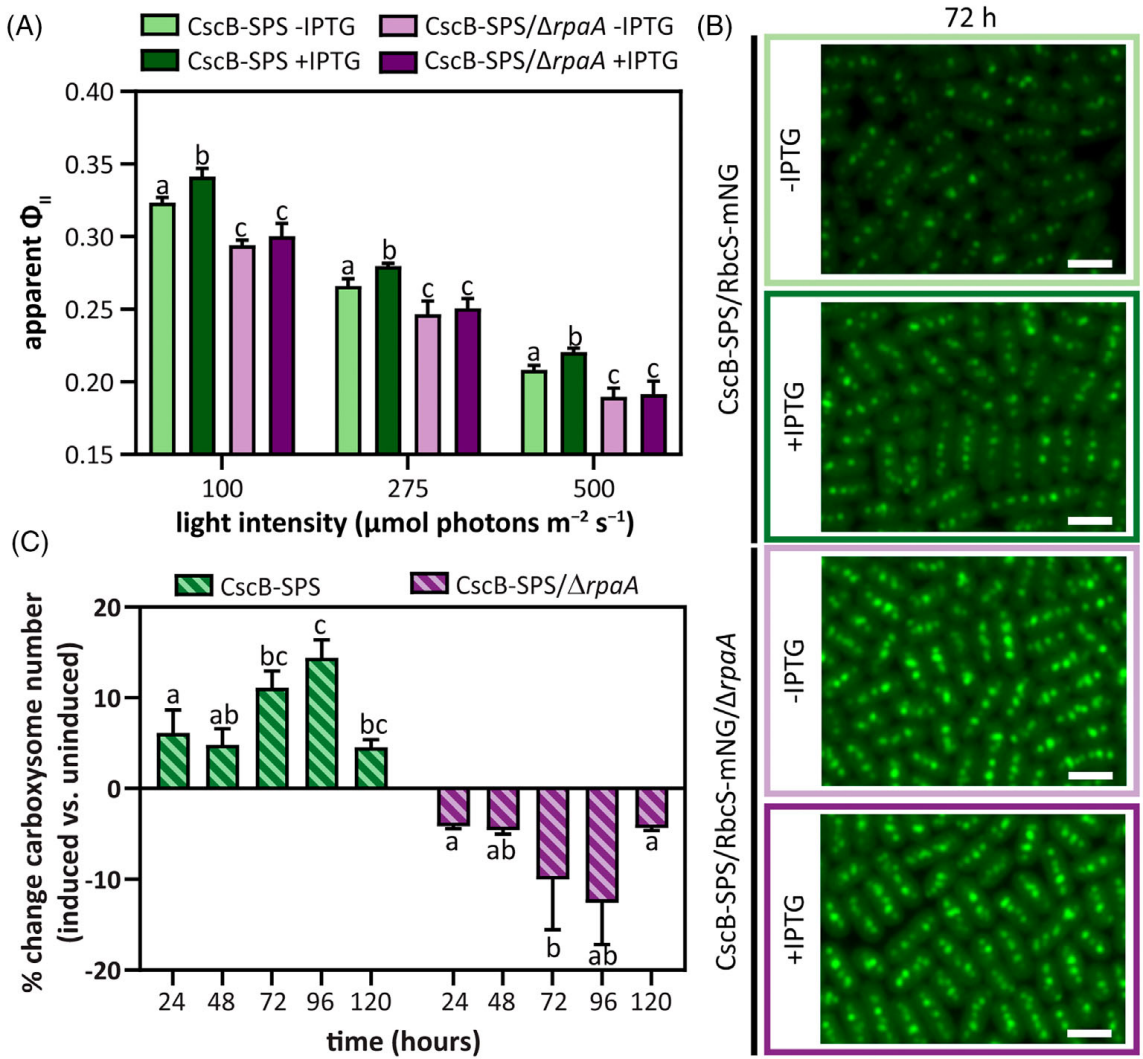
Acclimatory changes in the photosynthetic machinery following sucrose export are absent in Δ*rpaA*. (A) Apparent *Φ*_II_ values measured at three different light intensities 24 h after the induction of sucrose export in the strain CscB‐SPS^export^ in the presence/absence of RpaA. (B) Carboxysome number change (expressed as a percentage) after induction of sucrose export in the strain CscB‐SPS^export^ in the presence/absence of RpaA. (C) Carboxysomes at 72 h visualized via the RbcS‐mNG reporter in the strain CscB‐SPS^export^ in the presence/absence of RpaA. Scale bar: 2 μm. (A, B) Averages of ≥3 independent biological replicates are shown +SD. Significance was calculated by one‐way anova followed by Tukey's multiple comparison test. Data points labeled with different letters are significantly different (*P* < 0.05). (A–C) For these experiments, cells were grown at 32°C in 2% CO_2_ and with a light intensity of 150 μmol photons m^−2^ sec^−1^.

As discussed in the “Introduction” section, we recently implicated RpaA as a two‐component protein involved in the regulation of photosynthetic changes following activation of a heterologous sucrose secretion pathway (Santos‐Merino et al., 2024). To investigate the potential roles of RpaA in adapting photosynthesis and the CCM, we first validated the impact of loss‐of‐function mutants (Δ*rpaA*) in a strain expressing a carboxysome reporter (RbcS‐mNG) and bearing an inducible sucrose‐export pathway (CscB‐SPS^export^). We confirmed that the Δ*rpaA* strain lacked the characteristic rise in photosynthetic activity following activation of sucrose secretion, as measured by the apparent quantum efficiency of photosystem II (apparent *Φ*_II_; Figure 1A). Similarly, *rpaA* mutants failed to increase carboxysome number and size, as is typically observed following sucrose secretion (Singh et al., 2022) (Figure 1B,C; Figure S1). Importantly, the Δ*rpaA* background growth rate and other photosynthetic parameters appeared comparable to wildtype (WT) under constant light laboratory cultivation conditions, although Δ*rpaA* cell size was slightly diminished (Figure S2). These results are consistent with other recent reports that have demonstrated that Δ*rpaA* strains perform similarly to wildtype under conditions of constant light and high CO_2_; sometimes even exceeding wildtype growth (Diamond et al., 2017; Espinosa et al., 2015; Puszynska & O'Shea, 2017). Therefore, deletion of *rpaA* eliminates the enhancements in photosynthesis and increased carboxysome number typically observed in cells induced to export sucrose (Figure 1; Figure S1) but does not globally misregulate essential pathways required for growth and division under controlled laboratory conditions (Figure S2).

### Δ*rpaA* mutants exhibit growth arrest and dramatic carboxysome disassembly under mixotrophic conditions

**Figure 2:**
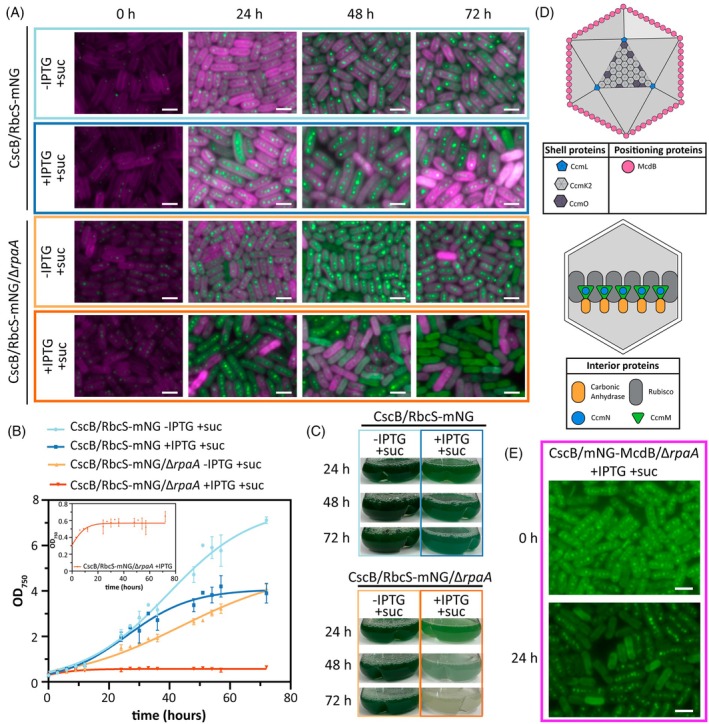
Mixotrophic conditions induce carboxysome breakdown and growth arrest in Δ*rpaA*. (A) Time course of carboxysome status under photoautotrophic and mixotrophic conditions in the strain CscB^import^ in the presence/absence of RpaA. Scale bar: 2 μm. (B) Growth curves of the strain CscB^import^ in the presence/absence of RpaA in photoautotrophic and mixotrophic conditions with a zoom in of the growth curve of CscB/RbcS‐mNG/Δ*rpaA*. Averages of ≥3 independent biological replicates are shown ±SD. (C) Appearance of the cultures of the strain CscB^import^ in the presence/absence of RpaA under photoautotrophic and mixotrophic conditions. (D) Cartoon illustration of internal and external carboxysome components. (E) Carboxysome status under mixotrophic conditions in the strain CscB^import^ in the presence/absence of RpaA by tracking mNG‐McdB. Scale bar: 2 μm. (A–C and E) For these experiments, cells were grown at 32°C in 2% CO_2_ and with a light intensity of 150 μmol photons m^−2^ sec^−1^.

As we and others have shown that carboxysome structure and composition are impacted by growth under mixotrophic conditions (Muth‐Pawlak et al., 2022; Singh et al., 2022), we next examined the impact of *rpaA* knockout on carboxysome restructuring under mixotrophy. While wild‐type *S. elongatus* is a strict photoautotroph, it has been previously engineered to grow mixotrophically with externally provided sugars when heterologous transporters are expressed (Kanno & Atsumi, 2017; McEwen et al., 2013; Singh et al., 2022). We encoded the sucrose permease gene under the IPTG‐inducible *trc* promoter to allow genetic control over sucrose import (CscB^import^) and supplemented the growth medium with sucrose. In a wild‐type background, mixotrophic growth mildly suppressed carboxysome number and increased the incidence of carboxysome clustering (Figure 2A), consistent with prior studies (Singh et al., 2022). By contrast, when CscB was expressed to allow the uptake of extracellular sucrose, Δ*rpaA* strains exhibited an immediate growth arrest and carboxysome reorganization followed by a dramatic disassembly of carboxysomes within 1–3 days of the onset of mixotrophic growth (Figure 2A–C; Figure S3). The loss of carboxysomal integrity appeared to be gradual, with some Δ*rpaA* cells displaying delocalized Rubisco signal throughout the cytosol in the population as early as 24 h, but only a fraction of cells exhibiting a complete loss of carboxysomal puncta (Figure S3a). The fraction of cells with diminished carboxysome puncta and increased cytosolic Rubisco localization increased over time, with nearly all cells lacking carboxysomes after 72 h of sucrose feeding (Figure 2A). We observed that cells displaying increased autofluorescence of chlorophyll *a* (possibly associated with impaired photosynthetic performance) were associated with a more rapid loss of carboxysome puncta over time (Figure 2A).

Loss of carboxysomes under mixotrophic growth conditions appears to be attributable to the dissolution of existing carboxysomes rather than failure to build new microcompartments because of the complete growth arrest observed (Figure 2B). To better assess the dynamics of carboxysome breakdown, we monitored the dynamics of maintenance of carboxysome distribution B (McdB) (Figure 2D), a ParB‐family protein involved in microcompartment positioning (MacCready et al., 2018). McdB utilizes a conserved motif to bind to the cytosolic‐facing side of bacterial microcompartment shell proteins, and it is therefore a relatively peripheral component of the carboxysome (Basalla et al., 2024). Within 24 h of the onset of mixotrophic growth in Δ*rpaA* cells, we observed delocalization of an endogenously tagged McdB‐mNG reporter from carboxysome puncta (Figure 2E; Figure S4a,b). A substantial fraction of cells exhibited complete loss of McdB localization, even while the Rubisco core of carboxysomes remained present in these cells. This suggests that carboxysome disassembly may initiate with components associated with the shell proteins and proceed inward. Delocalization of McdB was also observed in controls (Figure S4a,b, third vs. first rows), suggesting that the phenotype in Δ*rpaA* cells could be an exaggerated response to growth conditions where carboxysome components become delocalized in a WT background (Figure S4a,b, second and fourth rows). Consistent with the loss of the positional machinery, we observed that Rubisco puncta in mixotrophic Δ*rpaA* cells were mispositioned relative to one another or localized to the cell pole in certain cells (Figure S5). The localization of Rubisco to cell poles has been proposed to be an initiating step of carboxysome disassembly in prior studies (Hill et al., 2020).

Nitrogen deprivation is another well‐studied environmental stress condition that leads to rapid growth arrest and chlorosis (Collier & Grossman, 1992; Görl et al., 1998), analogous to the phenotypes we observed in Δ*rpaA* strains under mixotrophic conditions. To determine if carboxysome breakdown may be an under‐reported phenotype associated with other environmental stresses, we followed carboxysome integrity under nitrogen starvation conditions. Carboxysome puncta were retained in both WT and Δ*rpaA* cells, even under extended nitrogen limitation (Figure 6). The total number of carboxysomes per cell decreased in both backgrounds by 72 h of nitrogen starvation, but two or more RbcS‐mNG puncta were clearly retained in most cells. Nitrogen starvation in *S. elongatus* does not alter the expression of genes encoding for β‐carboxysome components, but it reduces protein synthesis (Choi et al., 2016). Therefore, it is plausible that reduced carboxysome number might be due to the inhibition of protein synthesis, an effect previously observed by the application of the protein synthesis inhibitor, lincomycin (Sun et al., 2016).

### Δ*rpaA* cells undergo a severe photosynthetic impairment under mixotrophy

**Figure 3:**
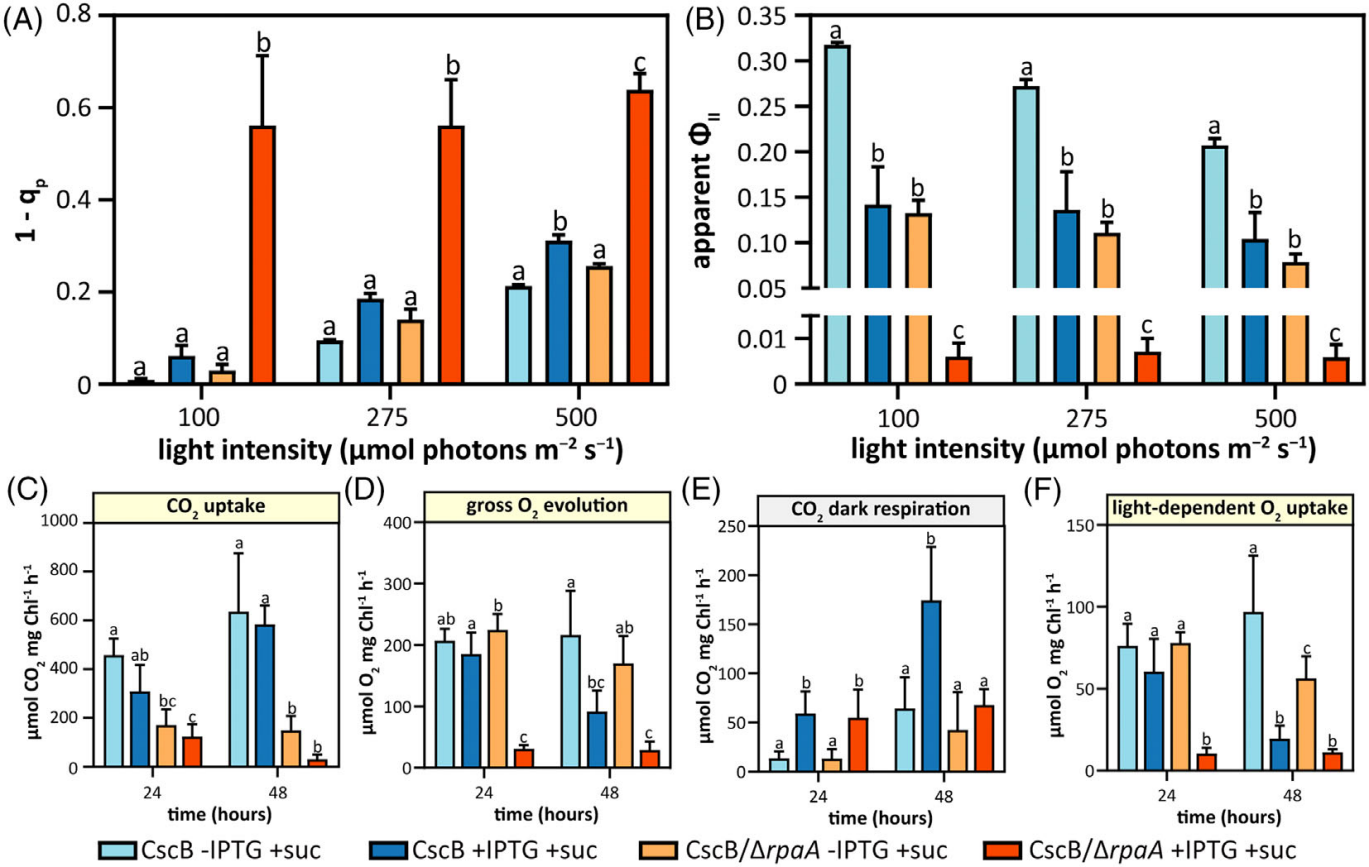
Mixotrophic growth conditions impair PSII activity and have different effects on the rate of O_2_ and CO_2_ fluxes. (A) 1 − *q*_p_ values. (B) Apparent *Φ*_II_ values. Quantification of the steady state flux rates shown in Figure S6(d–i) for (C) CO_2_ uptake, (D) gross O_2_ evolution, (E) CO_2_ dark respiration, and (F) light‐dependent O_2_ uptake for the strains CscB/RbcS‐mNG and CscB/RbcS‐mNG/Δ*rpaA*. CO_2_ exchange rates in the strains CscB/RbcS‐mNG and CscB/RbcS‐mNG/Δ*rpaA* under photoautotrophic and mixotrophic conditions. Averages of ≥3 independent biological replicates are shown +SD. Significance was calculated by one‐way anova followed by Tukey's multiple comparison test. Data points labeled with different letters are significantly different (*P* < 0.05). (A–F) For these experiments, cells were grown at 32°C in 2% CO_2_ and with a light intensity of 150 μmol photons m^−2^ sec^−1^.

The physiological characteristics of *rpaA* mutants under mixotrophic conditions (i.e., cell growth arrest, bleaching of pigments, and carboxysome breakdown) (Figure 2; Figures S3 and S4) suggest that photosynthetic processes may be misregulated (Calzadilla & Kirilovsky, 2020; Sunil et al., 2013). We directly assessed the photosynthetic performance of cultures grown under photoautotrophic and mixotrophic conditions via fluorimetry and spectroscopy. Estimates of the redox state of the PSII acceptor Q_A_ and, by inference, the PQ pool can be obtained by the photosynthetic parameters 1 − *q*_p_ and 1 − *q*_L_ (Kramer et al., 2004). In sucrose‐fed Δ*rpaA* strains, 1 − *q*_p_ and 1 − *q*_L_ were dramatically increased at all tested levels of illumination relative to modest changes in the sucrose‐fed control strain CscB/RbcS‐mNG (Figure 3A), indicating that the PQ pool was overreduced in the Δ*rpaA* lines under mixotrophic conditions at all irradiances (Figure 3A; Figure S7a). Similarly, the apparent *Φ*_II_ was reduced almost to zero under mixotrophic growth in Δ*rpaA* cultures, while it was only mildly suppressed in the reference control (Figure 3B). Other measured photosynthetic values were not as strongly impacted by sucrose feeding in either line, including the oxidation state of PSI or estimates of electron flux from cytochrome *b*_*6*_ *f* (Figure S7b,c). Overall, these results indicate that PSII and/or electron flux near PQ were the most strongly impacted.

To gain additional insight into photosynthetic fluxes in control and Δ*rpaA* lines, we turned to membrane inlet mass spectrometry (MIMS), which can disentangle CO_2_ and O_2_ gas exchange rates originating from photosynthetic and respiratory metabolism. Consistent with the sharp rise in *q*_P_ and decline in *Φ*_II_, we observed a rapid loss of photosynthetic CO_2_ uptake under both photoautotrophic and mixotrophic growth conditions in Δ*rpaA* cells (Figure 3C; Figure S7d,e), accompanied by a loss of O_2_ evolution only under mixotrophic conditions in the Δ*rpaA* strain (Figure 3D; Figure S7f,g). Continued CO_2_ respiration was observed in the Δ*rpaA* cells under dark conditions (Figure 3E; Figure S7d,e), providing direct evidence that the cells remain metabolically active during growth arrest (Figure 2). However, Δ*rpaA* cells exhibited a complete loss of O_2_ uptake induced by light under mixotrophic conditions (Figure 3E; Figure S7h,i), suggesting that reducing equivalents accumulated on the pETC are not consumed by alternative electron pathways that normally convey excess reductants to molecular oxygen (*i.e*., water–water cycles) (Nikkanen et al., 2021).

**Figure 4:**
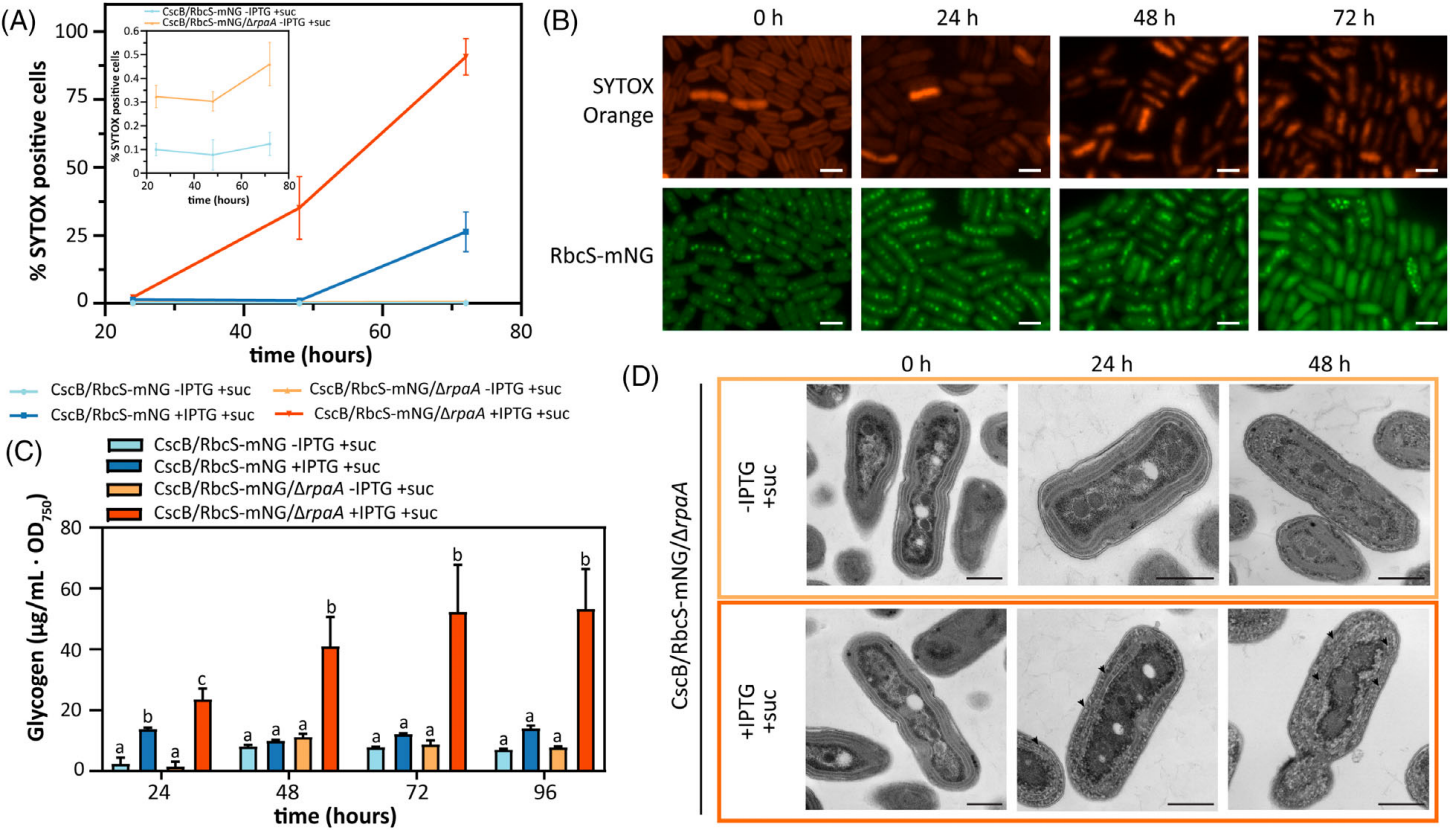
Carboxysome disassemble precedes cell death and is accompanied by glycogen accumulation. (A) Percentage of SYTOX Blue‐positive cells (dead cells) measured by flow cytometry under photoautotrophic and mixotrophic conditions, with detail of CscB/RbcS‐mNG and CscB/RbcS‐mNG/Δ*rpaA* under photoautotrophic conditions. Averages of ≥3 independent biological replicates are shown ±SD. (B) Time course of SYTOX Orange‐positive cells and carboxysomes by tracking RbcS‐mNG under mixotrophic conditions in the strain CscB^import^ in the absence of RpaA. Scale bar: 2 μm. (C) Quantification of glycogen per OD_750_ unit at different time points under photoautotrophic and mixotrophic conditions in the CscB strain in the presence and absence of RpaA. Averages of ≥3 independent biological replicates are shown +SD. Significance was calculated by one‐way anova followed by Tukey's multiple comparison test. Data points labeled with different letters are significantly different (*P* < 0.05). (D) Transmission electron microscopy of cells of the strain CscB^import^ in the absence of RpaA grown photoautotrophically and mixotrophically; black arrows indicate glycogen granules. Scale bar: 500 nm. (A–D) For these experiments, cells were grown at 32°C in 2% CO_2_ and with a light intensity of 150 μmol photons m^−2^ sec^−1^.

Given the severity of the photosynthetic impairment that mixotrophic conditions initiated in the Δ*rpaA* line, we used the vital dye SYTOX to confirm that cells exhibiting carboxysome breakdown remained viable. Fluorescence‐activated cell sorting (FACS) showed that cell viability began to be impaired 48 h under mixotrophic growth, with no viable cells after 72 h (Figure 4A; Figure S8). This indicated that initiation of carboxysome disassembly (i.e., evident at 24 h) precedes that of the loss of cell viability (between 48 and 72 h under mixotrophic growth). Fluorescence microscopy with SYTOX confirmed this interpretation, as cells that show disruption of carboxysomes remain SYTOX negative 24 and 48 h into the mixotrophic‐induced growth arrest (Figure 4B; Figure S5). To further evaluate the interplay between carboxysome breakdown and cell viability in Δ*rpaA* cells, we fed sucrose for 60 h (when nearly all Δ*rpaA* cells have no evident carboxysomes), then tracked recovery by returning cells to photoautotrophic growth (Figures S9 and S10). Following sucrose removal, Δ*rpaA* cells exited the growth arrest (<24 h), recovered pigmentation (24–48 h), and reassembled carboxysomes (24–72 h) (Figure S9a–e). Whereas growth of Δ*rpaA* cells immediately resumed when sucrose was removed (Figure S9d), recovery of cellular pigmentation and carboxysome puncta was delayed (Figure S9a–c). A further indication that growth‐arrested cells remained metabolically active was the greatly elevated ROS levels in sucrose‐fed Δ*rpaA* cultures; ROS levels gradually returned to the baseline of paired controls by 72 h following removal of sucrose (Figure S9e).

RpaA has recently been implicated in the regulation of central carbon metabolism and glycogen mobilization (Diamond et al., 2015, 2017; Puszynska & O'Shea, 2017; Santos‐Merino et al., 2024), so we examined the utilization of sucrose in Δ*rpaA* lines. We observed that sucrose feeding leads to glycogen accumulation in both WT and Δ*rpaA* backgrounds in the first 24 h. However, while the WT background restored normal glycogen levels by 48 h, Δ*rpaA* lines exhibited a continual accumulation of glycogen over time (Figure 4C). Indeed, glycogen hyperaccumulation in sucrose‐fed Δ*rpaA* cells was directly observable by electron microscopy, which revealed that glycogen bodies proliferated throughout the cytosol and within the thylakoid membranes (Figure 4D). Taken together, fluorescence kinetics, MIMS, vital dyes, and electron microscopy suggest that mixotrophic growth in Δ*rpaA* lines induces PSII impairments associated with overreduction of the PQ pool, arrests cell growth while maintaining metabolic activity, triggers abnormal levels of glycogen deposition, initiates carboxysome disassembly, and eventually can lead to cell death under prolonged exposure.

### PQ overreduction leads to H_2_O_2_ formation and carboxysome breakdown

A variety of stressors can lead to imbalances within the pETC, often leading to the activation of photoprotective mechanisms including alternative pathways to quench excess reductant to avoid the formation of ROS and photodamage (Pospisil, 2016). In the Δ*rpaA* mutant, we observe several signs that indicate an accumulation of electrons in the pETC, including a rise in the values of 1 − *q*_p_ and 1 − *q*_L_, a drop in the apparent *Φ*_II_ (Figure 3A,B; Figure S7a), and the loss of gross O_2_ evolution (Figure 3D,F; Figure S7f–i). Moreover, the suppression of light‐induced O_2_ uptake we observed in mixotrophic Δ*rpaA* strains (Figure 3F), together with unchanged P700 oxidation levels (Figure S7c), suggests that PQ oxidation via the cytochrome *b*_*6*_ *f* complex could be impaired, preventing electrons from reaching PSI (Roach & Krieger‐Liszkay, 2014).

**Figure 5:**
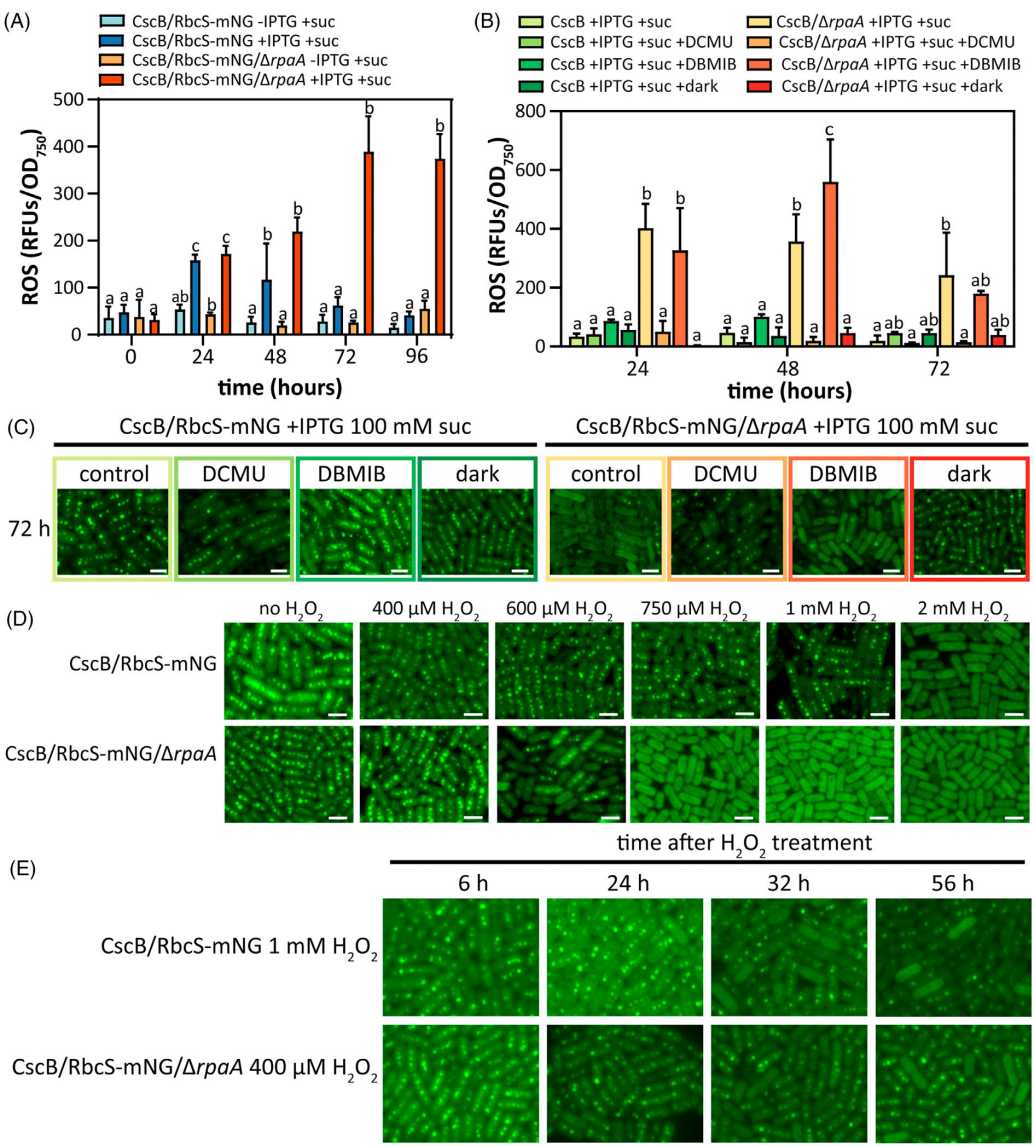
Reduction of the PQ pool leads to accumulation of ROS and carboxysome disassembly. Quantification of cellular ROS accumulation measured by H_2_DCFDA fluorescence at different time points (A) following activation of sucrose import, and (B) following the addition of photosynthesis inhibitors or growing the cells in darkness in sucrose feeding conditions in the CscB^import^ strain in the absence of RpaA. (C) Carboxysome status at 72 h in response to photosynthesis inhibitors or growing the cells in darkness in sucrose feeding conditions in the CscB^import^ strain in the presence/absence of RpaA by tracking RbcS‐mNG in the fluorescence microscope. Scale bar: 2 μm. (D) Carboxysome status after 24 h exposure to different concentrations of H_2_O_2_ in the CscB^import^ strain in the presence/absence of RpaA by tracking RbcS‐mNG. Scale bar: 2 μm. (E) Carboxysome status after exposure to 1 mm of H_2_O_2_ for CscB^import^ strain and 400 μm H_2_O_2_ for CscB^import^ strain in the absence of RpaA by tracking RbcS‐mNG. Scale bar: 2 μm. (A, B) Averages of ≥3 independent biological replicates are shown ±SD. Significance was calculated by one‐way anova followed by Tukey's multiple comparison test. Data points labeled with different letters are significantly different (*P* < 0.05). (A–E) For these experiments, cells were grown at 32°C in 2% CO_2_ and with a light intensity of 150 μmol photons m^−2^ sec^−1^.

Because of the indicators of potential imbalances in the pETC, we directly monitored ROS production in Δ*rpaA* cells and observed a sharp increase in ROS production peaking 72 h after initiation of mixotrophic conditions (Figure 5A). To further dissect the phenotype, we used chemical inhibitors and growth conditions to modulate the redox status of the pETC. Treatment of Δ*rpaA* cells with 3‐(3,4‐dichlorophenyl)‐1,1‐dimethylurea (DCMU), an inhibitor of PSII that blocks electron transport from Q_A_ to Q_B_ and thereby oxidizes the pETC at PQ and downstream electron carriers, decreased ROS production under mixotrophic conditions and prevented carboxysome breakdown (Figure 5A,C; Figure S11c). In parallel, sucrose feeding of Δ*rpaA* in the dark, where photosynthesis is inactive (Khorobrykh et al., 2020), also prevented both ROS production and preserved carboxysome integrity. By contrast, treatment with dibromothymoquinone (DBMIB), which is thought to block electron transfer from PQ to Cytochrome b_*6*_f, did not rescue sucrose‐fed Δ*rpaA* cells, which continued to strongly produce ROS and exhibit carboxysome breakdown (Figure 5A,C; Figure S11c). Darkness and DCMU treatment also recovered the loss in cell viability and pigmentation usually observed in mixotrophic Δ*rpaA* cells (Figure S11). Taken together, our results suggest that overreduction of PQ in Δ*rpaA* cultures is associated with ROS formation and carboxysome breakdown.

Hydrogen peroxide (H_2_O_2_) is a dominant form of ROS that has established signaling roles in plants (Mubarakshina & Ivanov, 2010), although any conserved regulatory function in cyanobacteria has not been clearly established (Latifi et al., 2009). We therefore tested the direct application of H_2_O_2_, observing that this treatment also impacted cellular pigmentation and carboxysome integrity in both WT and Δ*rpaA* strains (Figure 5D). Interestingly, the Δ*rpaA* mutant exhibited increased sensitivity to the external addition of H_2_O_2_ in terms of cell viability, pigmentation, and carboxysome integrity (Figure 5D; Figures S12 and S13). Acute treatment with H_2_O_2_ (2 mm H_2_O_2_ for WT or 750 μm H_2_O_2_ for Δ*rpaA*) led to a rapid bleaching and disassembly of most carboxysomes within the first 24 h. After acute H_2_O_2_ treatment under 2% CO_2_, those cells that still contained visible RbcS‐mNG puncta typically possessed only one or two carboxysomes, which were typically located in the pole of the cells (Figure S12d). This localization pattern has been previously reported as preceding carboxysome degradation (Hill et al., 2020) and the presence of these carboxysome‐like polar bodies has been associated with the lack of an effective shell structure (Rae et al., 2012). Yet, cell cultures do not always recover growth following acute H_2_O_2_ treatment, complicating the interpretation of these results. We therefore conducted further analysis using H_2_O_2_ treatments at concentrations where cell growth and pigmentation were unaffected (Figure S13a). Cells treated with subacute H_2_O_2_ (1 mm for WT or 400 μm for Δ*rpaA*) did not exhibit substantive changes in growth or pigmentation (Figure S13). However, in the hours immediately following subacute H_2_O_2_ treatment, subtle alterations in carboxysome number and RbcS‐mNG puncta organization were observed (Figure 5E). Crucially, at later time points (36–72 h post H_2_O_2_ exposure), carboxysome phenotypes appeared exacerbated, with many cells in the population displaying heterogeneity in carboxysome brightness, mispositioned/polar carboxysome puncta, too few carboxysomes, or no carboxysome puncta at all (Figure 5E; Figure S12). The carboxysome phenotypes at later time points (≥36 h) following subacute H_2_O_2_ treatment were less severe but were similar in character and timing to phenotypes observed under mixotrophic growth (>24 h).

### Physiological conditions that lead to ROS generation are associated with carboxysome rearrangement

**Figure 6:**
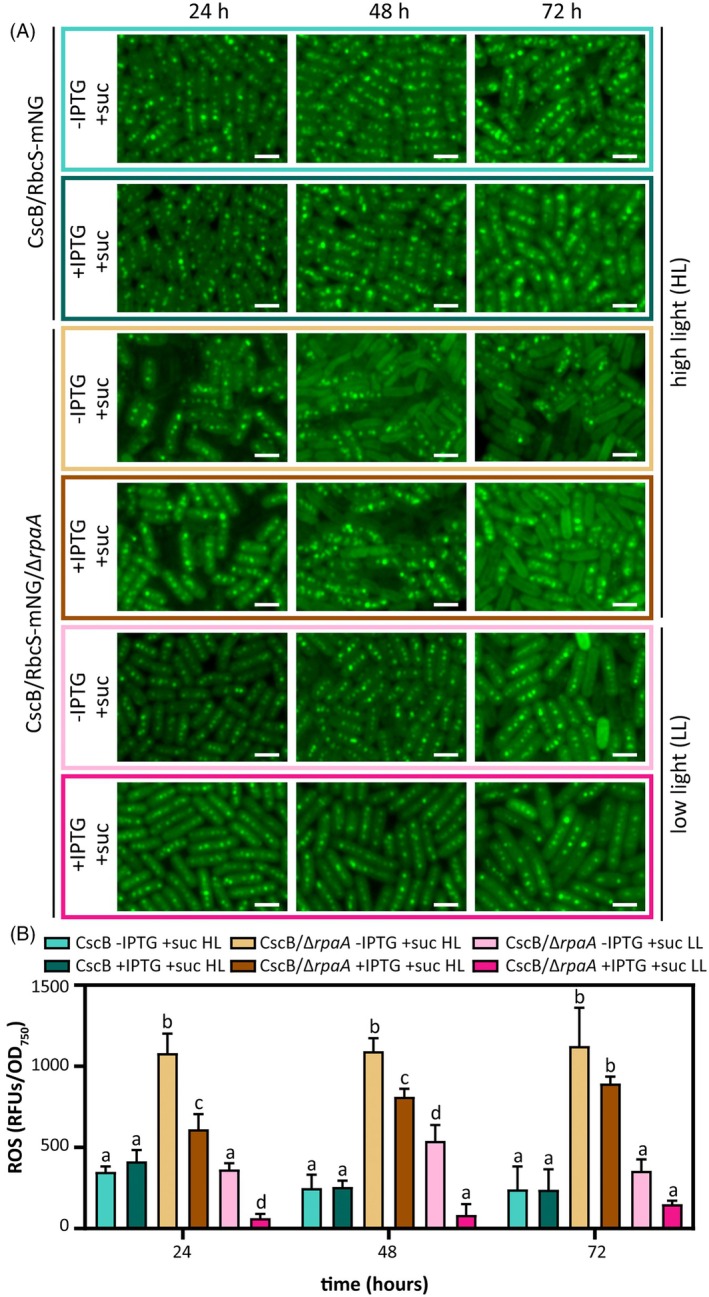
Carboxysome breakdown is delayed under ambient air conditions and low light intensity. (A) Carboxysome status in response to ambient air conditions under mixotrophic conditions for the strains CscB/RbcS‐mNG and CscB/RbcS‐mNG/Δ*rpaA* by tracking RbcS‐mNG. Scale bar: 2 μm. (B) Quantification of cellular ROS accumulation measured by H_2_DCFDA fluorescence at different time points following the transference to ambient air conditions during sucrose feeding conditions for the strains CscB/RbcS‐mNG and CscB/RbcS‐mNG/Δ*rpaA*. Averages of ≥3 independent biological replicates are shown +SD. Significance was calculated by one‐way anova followed by Tukey's multiple comparison test. Data points labeled with different letters are significantly different (*P* < 0.05). (A, B) For these experiments, cells were grown at 32°C in ambient air and with a light intensity of 150 μmol photons m^−2^ sec^−1^ (HL) or 17 μmol photons m^−2^ sec^−1^ (LL).

We next asked if other physiologically relevant environmental conditions are associated with carboxysome disassembly beyond mixotrophic growth, and we monitored any correlation with redox imbalance and ROS production. Light and inorganic carbon availability are two critical environmental factors strongly influencing cyanobacterial photosynthetic performance and ROS generation (Khorobrykh et al., 2020; Krieger‐Liszkay & Shimakawa, 2022). Therefore, we revisited WT and Δ*rpaA* cells under ambient CO_2_ conditions while varying both light intensity (HL, 150 μmol photons m^−2^ sec^−1^; LL, 17 μmol photons m^−2^ sec^−1^) and sucrose feeding. At ambient CO_2_ levels and HL, we found that Δ*rpaA* displayed substantial carboxysome breakdown even in the absence of sucrose feeding (Figure 6A, HL). Ambient CO_2_ alone was insufficient to induce carboxysome disassembly in Δ*rpaA* cells, as carboxysomes were well maintained in LL conditions both under photoautotrophic and mixotrophic conditions (Figure 6A, LL). We also detected an increase in ROS accumulation that was more pronounced under photoautotrophic conditions at both HL and LL in comparison to mixotrophic conditions (Figure 6B), though the absolute values of detected ROS were substantially higher across all tested cultures under air relative to 3% CO_2_ (Figure 5A). Intriguingly, mixotrophic growth partially rescued the loss of carboxysomes in Δ*rpaA* cells under ambient CO_2_ and HL (Figure 6A). In each of these cases, elevated ROS was strongly correlated with carboxysome disassembly (Figure 6B), as well as chlorophyll content and cell viability, although growth arrest was not observed under these conditions (Figures S14 and S15).

## DISCUSSION

Our findings suggest a novel regulatory link between photosynthetic redox status and carboxysome integrity, mediated in part by the transcription factor RpaA. Our results further emphasize emerging roles for RpaA in the regulation of cyanobacterial cellular redox balance and in key steps of central carbon metabolism. Furthermore, we identify new functions for RpaA in transitioning between different growth modes (e.g., photoautotrophic to mixotrophy), particularly with regard to cyanobacterial ROS production under dynamic conditions.

### Aberrant carboxysome regulation in Δ*rpaA* mutants is associated with ROS accumulation

**Figure 7:**
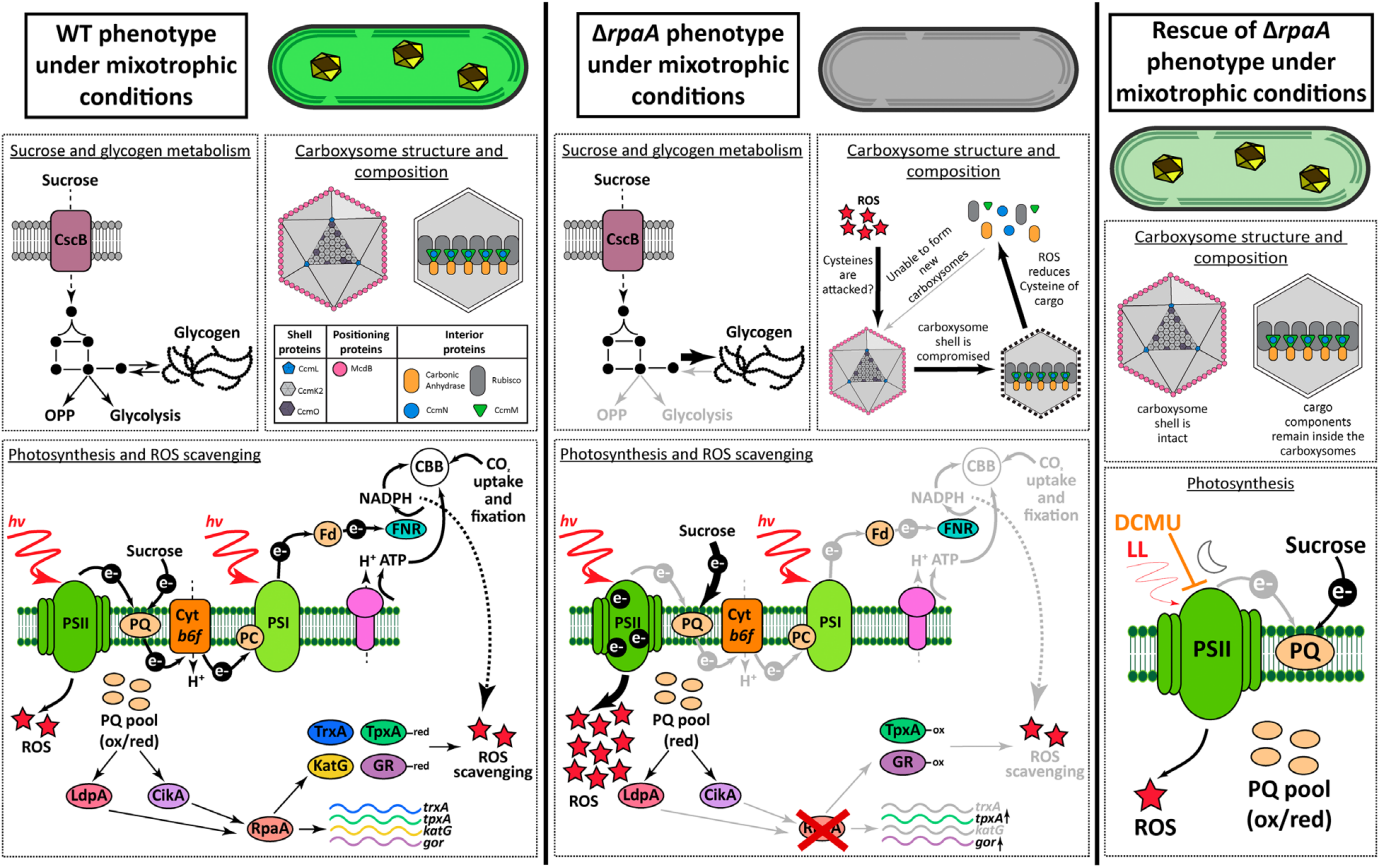
Proposed model of RpaA roles in *S. elongatus* under mixotrophic growth conditions. Under mixotrophic growth conditions, WT strain can metabolize sucrose to synthesize glycogen and relatively mild impacts on photosynthetic activity, PQ reduction, and carboxysome reorganization are observed (Singh et al., 2022). WT cells under mixotrophy show a transient increase in ROS levels initially but appear to have sufficient activity levels of scavenging systems to reduce ROS over time. In the absence of RpaA, sucrose is metabolized, and an overaccumulation of glycogen is observed (Figure 4C,D), perhaps in part to previous reports of downregulated transcription of genes responsible for glycogen breakdown (Puszynska & O'Shea, 2017). A dramatic decline in the apparent availability of oxidized PQ is observed in Δ*rpaA* under mixotrophy, contributing to severe impairment of PSII activity and an associated prolonged burst of ROS (Figures 3 and 5). It is possible that ROS that accumulates cannot be detoxified due to incomplete expression of scavenging enzymes, especially thioredoxin (Markson et al., 2013; Puszynska & O'Shea, 2017). Overaccumulation of oxidizing ROS may lead to impairment of carboxysome integrity via direct interaction with redox‐responsive cystine residues of structural components of carboxysomes. Finally, the integrity of the carboxysomes can be maintained in Δ*rpaA* by avoiding ROS formation and the reduction of the PQ pool under dark conditions, low light, or by using inhibitors of PSII (i.e., DCMU).

We find that Δ*rpaA* mutants display dramatic reorganization and disassembly of carboxysomes under conditions of PQ overreduction and associated ROS formation (Figures 2 and 5, 6, 7; Figures S3, S4, and S13). Under mixotrophy, Δ*rpaA* mutants exhibit a prolonged period of PQ overreduction and H_2_O_2_ generation, leading to near‐complete carboxysome breakdown. Notably, this breakdown is prevented by conditions that limit photosynthetic PQ reduction, including DCMU, low light, or darkness (Figures 5 and 7; Figure S11). While photosynthetic inhibitors (i.e., DCMU and DBMIB) are regularly utilized to manipulate the cyanobacterial pETC (Hihara et al., 2003; Khorobrykh et al., 2020; Kiss et al., 2009), they can have pleiotropic effects, particularly with longer periods of treatment (Schmitz et al., 1999; Schuurmans et al., 2014), so it is important that observed carboxysome phenotypes can be replicated with other environmental conditions. Similarly, carboxysomes can be reformed in cells after removal of the source of stress leading to pETC overreduction (Figure 2; Figures S3 and S9). Furthermore, direct H_2_O_2_ application initiates breakdown of carboxysomes *in vivo*, indicating this species of ROS is sufficient to elicit the phenotype (Figure 5D; Figure S12). Finally, Δ*rpaA* mutants also fail to make more subtle rearrangements under growth conditions known to modulate carboxysome morphology. Under steady state conditions, they show elevated Rubisco content and increased carboxysome number (Figure 1; Figure S1). Furthermore, the increases in carboxysome abundance, Rubisco content, and CO_2_ fixation observed upon activation of a heterologous sucrose sink (Ducat et al., 2012; Singh et al., 2022; Wang et al., 2023) are abolished in the Δ*rpaA* background (Figure 1B,C; Figure S1).

The carboxysome phenotype we observe is novel, as cyanobacteria typically exhibit constitutive assembly of these microcompartments. To our knowledge, carboxysome breakdown has been reported in literature in only two other contexts. First, in live‐cell imaging studies of cyanobacteria genetically engineered to gradually deplete structural components of the carboxysome, degrading carboxysomes were first mislocalized from the nucleoid to the cell pole prior to the dissolution of the Rubisco core (Hill et al., 2020), lacking an intact shell structure (Rae et al., 2012). This visualization of the carboxysome lifecycle parallels our observation of mispositioned, polar Rubisco puncta that precede carboxysome loss in Δ*rpaA* mutants (Figures 2, 5, and 6; Figures S3–S5, S11, and S12). This phenotype might also be also associated with the loss of shell components and the retention of procarboxysomes (Rubisco aggregates via CcmM) (Cameron et al., 2013). However, further characterization of these mispositioned polar puncta should be performed to identify the components remaining in these structures. Secondly, partial carboxysome disassembly has been documented in *Microcystis aeruginosa*, where the toxin microcystin binds Rubisco and disrupts its encapsulation, causing delocalization of Rubisco from carboxysomes to thylakoid‐associated aggregates (Barchewitz et al., 2019). Intriguingly, microcystin also sensitizes *M. aeruginosa* to H_2_O_2_ by inhibiting thioredoxin and peroxiredoxin (Alexova et al., 2016; Schuurmans et al., 2018), suggesting that ROS buildup also accompanies delocalization of Rubisco upon microcystin exposure.

The precise mechanism of carboxysome disassembly we observe in Δ*rpaA* cells remains unclear, although prior evidence suggests that H_2_O_2_‐induced oxidation may compromise carboxysome integrity. Many proteins contain redox‐reactive features, such as cysteine residues, that detect and transduce changes in cellular redox status during ROS accumulation (Anjum et al., 2015; Barford, 2004). These features are now recognized in several core components of cyanobacterial carboxysomes, highlighting a growing appreciation for redox regulation in carboxysome assembly (Borden & Savage, 2021; Huffine, 2025; Turnsek et al., 2024; Wang et al., 2019). More specifically, new carboxysomes have been shown to possess a reduced lumen which becomes more oxidized over the course of its maturation process (Chen et al., 2013). For instance, the scaffolding proteins CcmM and CcmN, as well as the cargo proteins Rubisco and carbonic anhydrase (CcaA), contain cysteines subject to redox control (Marcus et al., 2003; Price et al., 1992; Sun et al., 2021; Wang et al., 2019). Oxidation of CcmM cysteines reduces its affinity for Rubisco, disrupting the condensation step required for biogenesis (Cameron et al., 2013; Wang et al., 2019). Similarly, cysteine oxidation in RbcL and CcaA inhibits their enzymatic activity and, in the case of RbcL, promotes degradation (Marcus et al., 2003; Price et al., 1992). Rubisco itself may be oxidized and inhibited by ROS, consistent with the sharp decrease in CO_2_ assimilation (Figures 3C and 7; Figure S7d,e). Thus, elevated ROS in Δ*rpaA* mutants under mixotrophy (Figures 5A,B and 6B) may discourage new carboxysome biogenesis (Figure 7) until the level of oxidizing agents is reduced (Figure S9f).

One possible interpretation of our data is that PQ reduction and associated ROS formation are part of a regulatory mechanism cyanobacteria use to remodel carboxysomes in response to environmental changes. The size, number, and subunit composition of carboxysomes are known to correlate with light intensity and CO_2_ levels (Lucius & Hagemann, 2024; Rillema et al., 2021; Rohnke et al., 2018; Sun et al., 2016, 2019). In one particularly relevant example, a high‐light induced increase in carboxysome number was partially blocked by DCMU in *S. elongatus* (Sun et al., 2016). It is also well established that high light or low CO_2_ can overreduce the PQ pool and generate ROS (Lea‐Smith et al., 2013). Our data extend upon prior publications by suggesting PQ‐dependent carboxysome remodeling may be a function of ROS (potentially H_2_O_2_) derived from overreduction of this redox carrier, although we cannot exclude other potential sources of ROS (e.g., charge recombination in PSII) (Krieger‐Liszkay et al., 2008; Krieger‐Liszkay & Shimakawa, 2022). In this context, H_2_O_2_ – whether from photorespiration or direct application – was recently shown to modulate the algal pyrenoid, promoting tighter Rubisco aggregation and a more defined starch sheath (Neofotis et al., 2021). While speculative, our data imply H_2_O_2_ may similarly act in a signaling or regulatory role linking pETC redox status to CCM reorganization in cyanobacteria.

### RpaA functions balancing cellular redox and carbon partitioning

RpaA has established roles as a master regulator of cyanobacterial transcription (Markson et al., 2013), making it difficult to attribute the increased ROS production we observe in Δ*rpaA* mutants to a single cause or pathway. Nonetheless, recent reports highlight at least two key RpaA regulatory outputs closely related to our observations: modulation of redox balance and control of partitioning in central carbon metabolism (Diamond et al., 2015, 2017; Iijima et al., 2015; Johnson et al., 2024; Puszynska & O'Shea, 2017; Scheurer et al., 2021). One way in which Δ*rpaA* mutants may be susceptible to redox imbalance is that they are deficient in thioredoxin (TrxA) (Markson et al., 2013; Puszynska & O'Shea, 2017), which is required to reduce the cysteines in 2‐Cys peroxiredoxin (TpxA) (Mallén‐Ponce et al., 2021). TpxA is the main enzyme in *S. elongatus* responsible for detoxifying ROS, including H_2_O_2_, and has been previously shown to be upregulated under photomixotrophic growth conditions (Perelman et al., 2003; Tan et al., 2022). More directly, we have recently shown enrichment of TpxA and catalase‐peroxidase (KatG) in proximity labeling studies with RpaA (Santos‐Merino et al., 2024), indicating a possible direct RpaA interaction that would require additional study. Redox sensitivities evident in Δ*rpaA* mutants may be further exacerbated by altered metabolic partitioning, as in the case of the well‐described loss of viability of RpaA‐deficient cells when grown under light/dark cycles. This dark‐induced loss in viability is thought to be caused by a “redox crisis,” where Δ*rpaA* mutants fail both to route enough carbon towards glycogen storage during the day and exhibit reduced capacity to liberate carbon from glycogen at night; this leaves Δ*rpaA* cells deficient in NADPH for ROS detoxification in the dark (Diamond et al., 2017; Puszynska & O'Shea, 2017). Indeed, glycogen is an important metabolic buffer in many contexts, acting as a “sink” for photosynthetic metabolism during stress or nutrient deficiency and required to rapidly replenish carbohydrate intermediates to efficiently “reboot” the Calvin‐Benson‐Bassham (CBB) cycle during environmental fluctuations (Makowka et al., 2020; Shinde et al., 2020). Improper glycogen regulation may therefore lead to source/sink imbalances under a variety of contexts, which are documented to be tied to photoinhibition and ROS production in many green lineage species (Adams et al., 2014; Santos‐Merino, Singh, & Ducat, 2021).

An alternative interpretation based on established literature is that RpaA might have the capacity to more directly sense cellular redox status or the PQ pool. For instance, CikA is a circadian input kinase that directly senses quinone pool redox status and also regulates the phosphorylation status of RpaA (Ivleva et al., 2006; Kim et al., 2012). LdpA is another circadian protein that binds RpaA and has an iron‐sulfur cluster proposed to be modulated by cellular redox (Ivleva et al., 2005). Further reports connect RpaA with the redox state of the pETC through interactions with ferredoxin (Fd) and thioredoxin (TrxA) in experiments *in vitro* (Hanke et al., 2011; Kadowaki et al., 2015). Finally, redox‐responsive cysteines in RpaA have been shown to be modulated by TrxA, and the reduction status of these residues influences RpaA oligomeric state (Ibrahim et al., 2022). Given that the redox status of Cys65 of RpaA has recently been reported to undergo light‐dependent cycles *in vivo* (Johnson et al., 2024), it is feasible that RpaA functions may be more directly modified via interaction with redox partners. Future work will be required to disentangle the mechanism(s) by which RpaA is modulated by the redox status of the cell.

Taken together, we propose a provisional model in Figure 7 whereby RpaA plays a role in connecting cellular redox status, and especially the PQ pool, to carboxysome organization and function. An emerging theme across multiple studies implies that RpaA‐deficient cells are prone to dysregulation of energy balance when challenged to transition across a variety of environmental (e.g., day/night, high light) or metabolic (e.g., phototrophy/mixotrophy, carbon fixation/respiration) regimes. Furthermore, due to the roles for RpaA in regulating metabolic redox pools, detoxifying ROS, and/or controlling alternative pETC electron transport pathways, Δ*rpaA* mutants are likely primed to overproduce ROS during such transitions. We hypothesize that the prolonged burst of H_2_O_2_ we observe under PQ‐overreducing conditions may oxidize key structural components of the carboxysome, inhibiting new carboxysome biogenesis and destabilizing existing carboxysomes over time (Figure 7). Although distinct from previous reports of glycogen deficiency in Δ*rpaA* mutants (Puszynska & O'Shea, 2017), glycogen hyperaccumulation under mixotrophic conditions (Figure 4C,D) further underscores the misregulation of partitioning of central carbon metabolism in RpaA‐deficient lines.

Collectively, our data points to an additional regulatory role of the PQ pool as connected to the integrity and/or remodeling of the carbon fixation machinery under transitional periods where there may be a mismatch between the light and dark reactions. This control appears to involve RpaA, revealing a previously underappreciated function for the multi‐layered regulation of both PQ and RpaA in achieving energy balance in cyanobacteria.

## METHODS

### Strains and culture conditions

*S. elongatus* cultures were grown in BG11 medium supplemented with 1 g L^−1^ HEPES to a final pH of 8.3 with NaOH. Flasks were cultured in a Multitron incubator (Infors HT, Bottmingen, Switzerland) at 32°C under ambient air CO_2_ or supplemented with 2% CO_2_ with ~150 μmol photons m^−2^ sec^−1^ of light provided by Sylvania 15 W Gro‐Lux fluorescent bulbs and shaken at 150 rpm. For experiments under nitrogen starvation, cells were transferred into a medium lacking sodium nitrate (BG11_O_). Cultures were back‐diluted daily to an OD_750_ of 0.3 and acclimated to the medium/irradiance for at least 3 days prior to experiments or isopropyl‐β‐d‐thiogalactoside (IPTG) induction. Where appropriate, 1 mm IPTG was used to induce *cscB* and *sps* gene expression. Erythromycin (Em; 100 μg ml^−1^), chloramphenicol (Cm; 25 μg ml^−1^), and spectinomycin (Sp; 100 μg ml^−1^) were used to maintain *cscB‐sps*‐, *cscB*‐, and *rbcS‐mNG*‐containing cells, respectively. Kanamycin (Kn; 12.5 μg ml^−1^) was used to maintain the *rpaA* inactivation mutant Δ*rpaA*. In all cases, antibiotic selection was removed prior to conducting any of the reported experiments to minimize any unintended effects. When indicated, cultures were grown in the presence of photosynthesis inhibitors, including DCMU (20 μm) or DBMIB (10 μm). All strains used in this study are listed in Table 1.

**Table 1:** Cyanobacterial strains used in this study

Strain	Relevant genotype and phenotype	Plasmids used to generate this strain	Source or reference
WT	*S. elongatus* PCC 7942 wild type strain		PCC
WT/RbcS‐mNG	*S. elongatus* PCC 7942 wild type strain with P_*rbcS*_::*rbcS‐mNG* integrated at NS1; Sp^r^	pRbcS‐mNG	This work
WT/RbcS‐mNG/Δ*rpaA*	*S. elongatus* PCC 7942 wild type strain with P_*rbcS*_::*rbcS‐mNG* integrated at NS1, and with Δ*rpaA*::*aacC1*; Sp^r^ Gm^r^	pRbcS‐mNG pMD19‐T‐0095	This work
CscB‐SPS^export^	*S. elongatus* PCC 7942 with P_*trc*_::*cscB* and P_*trc*_::*sps* integrated at NS3; Em^r^		Santos‐Merino et al. (2024)
CscB‐SPS^export^/Δ*rpaA*	*S. elongatus* PCC 7942 with P_*trc*_::*cscB* and P_*trc*_::*sps* integrated at NS3, and with Δ*rpaA*::*aph1*; Em^r^ Kn^r^		Santos‐Merino et al. (2024)
CscB‐SPS^export^/RbcS‐mNG	*S. elongatus* PCC 7942 with P_*trc*_::*cscB* and P_*trc*_::*sps* integrated at NS3, and with P_*rbcS*_::*rbcS‐mNG* integrated at NS1; Em^r^ Sp^r^	pRbcS‐mNG	This work
CscB‐SPS^export^/RbcS‐mNG/Δ*rpaA*	*S. elongatus* PCC 7942 with P_*trc*_::*cscB* and P_*trc*_::*sps* integrated at NS3, with P_*rbcS*_::*rbcS‐mNG* integrated at NS1, and with Δ*rpaA*::*aph1*; Em^r^ Sp^r^ Kn^r^	pRbcS‐mNG pMD19‐T‐0095	This work
CscB^import^	*S. elongatus* PCC 7942 with P_*trc*_::*cscB* integrated at NS3; Cm^r^		Ducat et al. (2012)
CscB^import^/RbcS‐mNG	*S. elongatus* PCC 7942 with P_*trc*_::*cscB* integrated at NS3, and with P_*rbcS*_::*rbcS‐mNG* integrated at NS1; Cm^r^ Sp^r^	pRbcS‐mNG	This work
CscB^import^/RbcS‐mNG/Δ*rpaA*	*S. elongatus* PCC 7942 with P_*trc*_::*cscB* integrated at NS3, with P_*rbcS*_::*rbcS‐mNG* integrated at NS1, and with Δ*rpaA*::*aph1*; Cm^r^ Sp^r^ Kn^r^	pRbcS‐mNG pMD19‐T‐0095	This work
CscB^import^/RbcS‐mNG	*S. elongatus* PCC 7942 with P_*trc*_::*cscB* integrated at NS3, and with the native copy of *mcdB* replaced with *mNG*‐*rbcS*; Cm^r^ Kn^r^	pmNG‐McdB	This work
CscB^import^/mNG‐McdB/Δ*rpaA*	*S. elongatus* PCC 7942 with P_*trc*_::*cscB* integrated at NS3, with the native copy of *mcdB* replaced with *mNG*‐*rbcS*, and with Δ*rpaA*::*aacC1*; Cm^r^ Kn^r^ Gm^r^	pmNG‐McdB pV0120‐0095	This work
CscB^import^/mNG‐McdB/RbcS‐mTQ/Δ*rpaA*	*S. elongatus* PCC 7942 with P_*trc*_::*cscB* integrated at NS3, with the native copy of *mcdB* replaced with *mNG*‐*rbcS*, with P_*rbcS*_::*rbcS‐mTQ* integrated at NS1, and with Δ*rpaA*::*aacC1*; Cm^r^ Kn^r^ Sp^r^ Gm^r^	pmNG‐McdB pRbcS‐mTQ pV0120‐0095	This work

Cm^r^, chloramphenicol resistance; Em^r^, erythromycin resistance; Kn^r^, kanamycin resistance; mTQ, mTurquoise; NS1, neutral site 1; NS3, neutral site 3; PCC, Pasteur culture collection; Sp^r^, spectinomycin resistance.

### Strain construction

*S. elongatus* with genomically integrated copies of *cscB* and *sps* under an IPTG‐inducible promoter was previously obtained (Santos‐Merino et al., 2024), and *S. elongatus* with genomically integrated copies of *cscB* under an IPTG‐inducible promoter was previously described (Ducat et al., 2012). The genomic loci encoding the *rpaA* gene were disrupted by inserting a kanamycin resistance cassette (Qiao et al., 2019). To allow visualization of changes in carboxysome organization, we integrated a fluorescent reporter fused to the small subunit of Rubisco (RbcS‐mNG) in NS1 by modifying a plasmid previously published (Sakkos et al., 2021). Plasmid details are reported in Table 2.

**Table 2:** Plasmids used in this study

Plasmid	Relevant genotype and phenotype	Source or reference
pAM2314‐RbcS‐mNG	pAM2314 with P_*rbcLS*_::*rbcS* fused to mNG for neutral site 1 integration; Cm^r^	Sakkos et al. (2021)
pRbcS‐mNG	Derivative of pAM2314‐RbcS‐mNG modified to replace *cat* gene conferring chloramphenicol resistance by *aadA* gene conferring spectinomycin resistance; Sp^r^	This work
pMD19‐T‐0095	Plasmid to replace the *rpaA* gene with a kanamycin resistance cassette (*aph1*); Ap^r^ Kn^r^	Qiao et al. (2019)
pV0120‐0095	Plasmid to replace the *rpaA* gene with a gentamicin resistance cassette (*aacC1*); Ap^r^ Gm^r^	This work
pmNG‐McdB	Plasmid to replace the endogenous copy of *mcdB* by a version fused to mNG; Kn^r^	MacCready et al. (2018)
pRbcS‐mTQ	Plasmid with P_*rbcLS*_::*rbcS* fused to mTQ for neutral site 1 integration; Sp^r^	This work

Ap^r^, ampicillin resistance; Cm^r^, chloramphenicol resistance; Kn^r^, kanamycin resistance; mTQ, mTurquoise; Sp^r^, spectinomycin resistance.

### Sucrose quantification

Secreted sucrose was quantified from supernatants using the Sucrose/d‐Glucose Assay Kit (K‐SUCGL; Megazyme, Bray, Ireland).

### Pigment determination

Chl*a* was extracted from cell pellets by incubation in 100% methanol for 30 min at 4°C. Chl*a* concentration was estimated by the spectrophotometric method described previously (Porra et al., 1989).

### Fluorescence measurements

Apparent quantum yield of PSII (*Φ*_II_) measurements was performed on a custom‐built fluorimeter/spectrophotometer as described previously (Santos‐Merino, Torrado, et al., 2021). Briefly, samples containing cyanobacteria (2.5 μg ml^−1^ chlorophyll) resuspended in fresh medium sparged with 2% CO_2_ in air were dark‐adapted for 3 min before measuring. The apparent quantum yield of photosystem II (*Φ*_II_), $\left(F_{M}^{\prime }-F_{S}\right)/\left(F_{M}^{\prime }\right)$, and the coefficients of photochemical quenching 1 − *q*_p_, $\left[1-\left(F_{M}^{\prime }-F_{S}\right)/\left(F_{M}^{\prime }-F_{0}^{\prime }\right)\right]$ and 1 – *q*_L_, $\left[1-\left(F_{q}^{\prime }-F_{v}^{\prime }\right)/\left(F_{0}^{\prime }-F_{S}\right)\right]$, were measured using a 1.5 sec saturating pulses of actinic light (~5000 μmol photons m^−2^ sec^−1^).

### PSI absorbance changes

To evaluate P700 redox changes, samples were monitored semi‐simultaneously with fluorescence measurements, using the instrument described in Hall et al. (2013) by measuring absorbance changes at about 703 nm. The measuring beam was generated by a pulsed LED (720 nm peak emission, Rebel LUXEON Far Red) filtered with a 5 nm bandpass filter centered at 700 nm, resulting in a measured emission peak at approximately 703 nm (Abramson et al., 2016). The signals were detected with a photodiode filtered with a Schott RG‐695 filter to block actinic light (Hall et al., 2013). Samples containing cyanobacteria cells were prepared by resuspending cells in fresh medium to a concentration of 5 μg ml^−1^ chlorophyll and sparged with 2% CO_2_ in air. The samples were dark‐adapted for 3 min before starting measurements. The percentage of oxidized P700 was calculated as [(*P*_ox_ − *P*_ss_)/(*P*_red_ − *P*_ox_)] × 100, where *P*_ox_ was the maximum extent of P700 absorbance signal induced by a saturating pulse of light during ∼0.5 sec (~5000 μmol photons m^−2^ sec^−1^); *P*_ss_, taken to be the fraction of P700^+^ in under steady state illumination, estimated by the extent of P700 absorbance signal induced by a short dark interval, and assuming that P700 reaches full reduction; and *P*_red_ is the level of P700 reduced in the dark. PSI traces were normalized to the last point of the steady state level of oxidation (*P*_ss_).

### Dark‐interval relaxation kinetics (DIRK) absorbance changes

Steady state levels of photooxidized P700 (P700^+^) were estimated by DIRK analysis (Sacksteder & Kramer, 2000) after 21 sec of actinic illumination at three different intensities (100, 275, and 500 μmol photons m^−2^ sec^−1^). Samples containing cyanobacteria (2.5 μg ml^−1^ chlorophyll) were resuspended in fresh medium containing 2% CO_2_ and were dark‐adapted for 3 min before measuring. The half‐time of P700^+^ re‐reduction (Tau) was measured from the absorbance change at 703 nm (Δ*A*_703_) during a dark interval of 2100 msec. Tau was calculated by the monotonic decay kinetic of Δ*A*_703_ produced after extinguishing the actinic light (Baker et al., 2007).

### Microscopy and image analysis

All live‐cell microscopy was performed on cells in exponential growth by centrifuging 2 ml of culture at 10 000 ***g*** for 5 min, resuspending into 80 μl of BG11, and transferring a 2 μl aliquot to a 3% agarose pad. The cells were allowed to briefly equilibrate and be absorbed by the agarose (≥10 min) before the pad was placed onto a #1.5 glass coverslip for imaging. Images were captured using a Zeiss Axio Observer D1 inverted microscope equipped with an Axiocam 503 mono camera and a Zeiss Plan Apochromat 63× 1.4 NA oil‐immersion lens.

Image analysis was done in OMERO (Allan et al., 2012) and Python 3. Cell segmentation was conducted using Cellpose (Stringer et al., 2021). Mean fluorescence intensity plots were generated as previously described (Sakkos et al., 2021). Briefly, cells were segmented using the chlorophyll autofluorescence channel, rotated such that the medial axis was horizontal, rescaled to ensure consistent boundaries, and the RbcS‐mNG pixel intensity was averaged from each cell in the collection of images from its respective induction condition and time point. Foci locations were determined with a peak‐finding algorithm using the Python package Photutils (Bradley et al., 2016).

### Cellular viability

Cellular death was quantified using SYTOX Blue (1 mm in DMSO; Invitrogen, Carlsbad, CA, USA; S34857) on a flow cytometer or SYTOX Orange (250 μM in DMSO; Invitrogen; S34861) and visualized by microscopy. SYTOX dyes are nucleic‐acid‐specific stains that are unable to reach the intracellular space due to the intact cell membrane of a non‐damaged cell. At each time point, 1 μl of the SYTOX blue working stock was added to 1 ml of culture (final concentration of 1 μm for SYTOX Blue or 250 nm for SYTOX Orange) and incubated in the dark at room temperature for 15 min. Heat‐treated cyanobacterial cells were used as a positive control for dead cells. After incubation, 200 μl aliquots of the cultures were transferred to a 96‐well plate to measure viability in the flow cytometer. Samples were collected on a 4‐laser Attune CytPix with a CytKick Max Autosampler Software 6.2.0 (Invitrogen). The following optical configuration was used for each fluorophore (excitation|emission): SYTOX Blue [405 nm|450/40] (BL1‐A, 305 V), Chlorophyll [405 nm|660/20] (486 V). Cyanobacterial samples were gated using FSC (400 V) and SSC (250 V) to distinguish the singlet population and with the chlorophyll to remove debris and noise; more than 10 000 cells were measured per sample type unless stated otherwise. Gating regions representing both intact, SYTOX‐negative and membrane‐damaged, SYTOX‐positive cells were created in two‐dimensional dot plots (forward side scatter vs. blue fluorescence). The raw data were analyzed using the FCS Express 7 software (De Novo Software, Pasadena, CA, USA). For the microscope images, samples were processed following a protocol described in Section “Microscopy and image analysis”.

### Membrane inlet mass spectrometry (MIMS)

Online measurements of gas exchange were monitored using a mass spectrometer (model Prima PRO; Thermo Scientific, Waltham, MA, USA). The membrane inlet system, consisting of a modified DW1 oxygen electrode chamber (Hansatech Instruments Ltd., Norfolk, UK) water‐jacketed thermoregulated at 30°C, was attached to the vacuum line of a mass spectrometer through a thin gas‐permeable PTFE membrane (0.0125 mm) sealing the bottom of the chamber. ^18^O_2_ (isotope purity >98%; CK Gas Products Ltd., Newtown Unthank, UK) tracing was used to discriminate O_2_ uptake and O_2_ production by PSII. ^16^O_2_ (*m/z* 32), ^18^O_2_ (*m/z* 36), and CO_2_ (*m/z* 44) were recorded with a time resolution of around 4 sec. Samples were evenly mixed by constant stirring using a cross‐shaped magnetic stirrer. A 2 ml aliquot of a cell suspension (10 μg ml^−1^ Chl*a*) was placed in the measuring chamber, and prior to the measurement, cells were supplemented with ^18^O_2_ at an equivalent concentration to ^16^O_2_ and with 1.5 mm NaHCO_3_. Then, samples were measured for 5 min in darkness to record oxygen consumption caused by respiration. Following this period, actinic light (500 μmol photons m^−2^ sec^−1^) was applied via a 150‐W, 21‐V EKE quartz halogen‐powered fiber optic illuminator (Fiber‐Lite DC‐950; Dolan‐Jenner, Boxborough, MA, USA). Gas‐exchange kinetics and rates were determined according to Beckmann et al. (2009). Final Chl*a* concentration, determined spectrophotometrically in 100% methanol according to Porra et al. (1989), was conducted at the completion of each measurement for standardizing the calculated gas exchange rates.

### Determination of intracellular glycogen content

Glycogen content was determined as described previously with minor modifications (Gründel et al., 2012). Cyanobacterial culture aliquots (2 ml) were pelleted down by centrifuging at 5000 ***g*** for 10 min. Pellets were flash‐frozen in liquid nitrogen and were stored at −80°C until extraction. For isolation of glycogen, the pellets were resuspended in 200 μl 30% (w/v) KOH and incubated in a heat block at 95°C for 2 h. Samples were cooled down on ice. Complete precipitation of glycogen was achieved by the addition of 600 μl of cold absolute ethanol and overnight incubation at −20°C. The precipitated glycogen was recovered by centrifugation at 17 000 ***g*** for 15 min at 4°C. The supernatant was removed, and the glycogen pellets were dried for 40 min at 60°C using a SpeedVac. The precipitated glycogen was resuspended in 200 μl of miliQ H_2_O by vortexing. The homogeneous samples were quantified using the EnzyChrome glycogen assay kit (BioAssay Systems, Woburn, MA, USA; E2GN‐100) according to the manufacturer's instructions.

### Transmission electron microscopy

At each time point, 2 ml samples were prepared by diluting cultures to a final OD_750_ of 1.5. Cells were pelleted and fixed overnight at 4°C with 2% glutaraldehyde/2% paraformaldehyde in phosphate buffer (pH 7.4), suspended in a 2% agarose bead, and cut into ~1 mm cubes. Following three washes with 0.1 m sodium cacodylate buffer, cells were suspended in 1% osmium tetroxide/1.5% potassium ferrocyanide and incubated overnight at 4°C. After incubation, cells were washed with HPLC‐quality H_2_O until they appeared clear. Cells were then suspended in 1% uranyl acetate and microwaved for 2 min using a MS‐9000 Laboratory Microwave Oven (Electron Microscopy Science, Hatfield, PA, USA), decanted, and washed until clear. Cells were dehydrated in an increasing acetone series (microwave 2 min) and then embedded in Spurr's resin (25% increments for 10 min each at 25°C). A final overnight incubation at room temperature in Spurr's resin was done; then, cells were embedded in blocks that were polymerized by incubation at 60°C for 3 days. Thin sections of approximately 50 nm were obtained using an MYX ultramicrotome (RMC Products), post‐stained with 1% uranyl acetate and Reynolds lead citrate, and visualized on a JEM 100CX II transmission electron microscope (JEOL,Tokyo, Japan) equipped with an Orius SC200‐830 CCD camera (Gatan, Pleasanton, CA, USA).

### Quantification of reactive oxygen species (ROS)

ROS were quantified by using the fluorescent marker H_2_DCFDA (2′,7′‐dichlorodihydrofluorescein diacetate; Invitrogen; D399) as previously reported (Diamond et al., 2017). Briefly, 2 ml of the cultures were collected and split into 1‐ml aliquots. H_2_DCFDA was added to one sample at a final concentration of 5 μm. Tubes were protected from light and shaken at 30°C for 30 min. After incubation, 200 μl of each tube was added to a separate well in a 96‐well plate. The fluorescent product 2′,7′‐dichlorofluorescein (DCF) was monitored via a microplate reader (excitation 480 nm, emission 520 nm; SpectraMax M2 microplate reader by Molecular Devices, San Jose, CA, USA). Untreated‐sample background fluorescence was then subtracted from treated‐sample fluorescence values, and fluorescence data were normalized to OD_750_ of each sample.

### Determination of growth of cyanobacterial cultures in the presence of hydrogen peroxide

To determine the growth of the different cyanobacterial strains in the presence of H_2_O_2_, cell cultures were adjusted to an OD_750_ of 0.3. Hydrogen peroxide, at final concentrations ranging from 50 μm to 2 mm, was added to 1.5‐ml aliquots of cultures in separate wells of a 24‐well plate or to 50‐ml flasks with cultures. Images of the plates, as well as fluorescence microscopy images, were taken after a 24‐h incubation under standard growth conditions.

### Statistical analysis

Recorded measurements are represented as mean values, with error bars expressing the SD of *n* ≥ 3 biological replicate experiments, as indicated. The significance of differences between groups was evaluated by one‐way anova followed by Tukey's multiple comparison test or by an unpaired Student's *t*‐test. Statistical analyses were carried out using GraphPad Prism software (GraphPad Software Inc., San Diego, CA, USA). Differences were considered statistically significant at *P* < 0.05.

## AUTHOR CONTRIBUTIONS

MS‐M and DCD conceived the project. MS‐M, LN, and EJK performed experiments and analyzed data. MS‐M and DCD wrote the manuscript with edits from LN, EJK, and YA. All authors read and approved the final manuscript.

## CONFLICT OF INTEREST

The authors declare no competing interests.

## Supporting information


**Figure S1.** Δ*rpaA* mutants do not reorganize carboxysomes in response to sucrose export.
**Figure S2.** Knockout of RpaA does not globally disrupt cell physiology under constant light.
**Figure S3.** Time‐course of carboxysome disassembly in the CscB/RbcS‐mNG/Δ*rpaA* strain under mixotrophic conditions.
**Figure S4.** The outer carboxysome component McdB is delocalized prior to the loss of core rubisco puncta following the onset of mixotrophic conditions.
**Figure S5.** Mobilization of carboxysome to the cell poles and carboxysome breakdown happens prior cell death.
**Figure S6.** Nitrogen starvation does not induce carboxysome breakdown.
**Figure S7.** Changes in photosynthesis and O_2_ and CO_2_ fluxes associated with mixotrophic conditions in Δ*rpaA*.
**Figure S8.** Dot plots of SYTOX blue flow cytometry revealed a negative effect of sucrose feeding in the Δ*rpaA* mutant.
**Figure S9.** Sucrose removal from media allows Δ*rpaA* cells to recover carboxysomes, viability, and growth and reduces ROS.
**Figure S10.** Dot plots of SYTOX blue flow cytometry revealed the recovery of the viability in the Δ*rpaA* mutant after sucrose removal from the media.
**Figure S11.** Effect of photosynthesis inhibitors and darkness treatment in the population viability, growth, carboxysomes, chlorophyll, and culture aspect.
**Figure S12.** Effects of H_2_O_2_ treatment.
**Figure S13.** Effect of sublethal concentrations of H_2_O_2_ in the chlorophyll and culture aspect.
**Figure S14.** Effect of ambient air conditions on chlorophyll, ROS levels, growth, and viability.
**Figure S15.** Dot plots of SYTOX blue flow cytometry after CO_2_ down‐shift revealed a negative in the viability of the Δ*rpaA* mutant even in the absence of sucrose feeding.

## Data Availability

The datasets analyzed during the current study are available from the corresponding author upon reasonable request.
